# Mechanistic study of silica nanoparticles on the size-dependent retinal toxicity in vitro and in vivo

**DOI:** 10.1186/s12951-022-01326-8

**Published:** 2022-03-19

**Authors:** Zhuhong Zhang, Laien Zhao, Yuanyuan Ma, Jia Liu, Yanmei Huang, Xiaoxuan Fu, Shengjun Peng, Xiaojie Wang, Yun Yang, Xiaoyan Zhang, Wanru Ding, Jinguo Yu, Yanping Zhu, Hua Yan, Shubin Yang

**Affiliations:** 1 School of Pharmacy, Key Laboratory of Molecular Pharmacology and Drug Evaluation (Yantai University), Ministry of Education, Collaborative Innovation Center of Advanced Drug Delivery System and Biotech Drugs in Universities of Shandong, Yantai University, Yantai, 264005 People’s Republic of China; 2 Department of Ophthalmology, Tianjin Medical University General Hospital, Tianjin, 300052 People’s Republic of China; 3 School of Chemistry and Chemical Engineering, Yantai University, Yantai, 264005 People’s Republic of China

**Keywords:** SiO_2_ NPs, Retinal toxicity, ROS, Ganglion cell degeneration, Glial cell activation, Inflammation

## Abstract

**Background:**

Silica nanoparticles (SiO_2_ NPs) are extensively applied in the biomedical field. The increasing medical application of SiO_2_ NPs has raised concerns about their safety. However, studies on SiO_2_ NP-induced retinal toxicity are lacking.

**Methods:**

We investigated the retinal toxicity of SiO_2_ NPs with different sizes (15 and 50 nm) in vitro and in vivo along with the underlying mechanisms. The cytotoxicity of SiO_2_ NPs with different sizes was assessed in R28 human retinal precursor cells by determining the ATP content and LDH release. The cell morphologies and nanoparticle distributions in the cells were analyzed by phase-contrast microscopy and transmission electron microscopy, respectively. The mitochondrial membrane potential was examined by confocal laser scanning microscopy. The retinal toxicity induced by SiO_2_ NPs in vivo was examined by immunohistochemical analysis. To further investigate the mechanism of retinal toxicity induced by SiO_2_ NPs, reactive oxygen species (ROS) generation, glial cell activation and inflammation were monitored.

**Results:**

The 15-nm SiO_2_ NPs were found to have higher cytotoxicity than the larger NPs. Notably, the 15-nm SiO_2_ NPs induced retinal toxicity in vivo, as demonstrated by increased cell death in the retina, TUNEL-stained retinal cells, retinal ganglion cell degeneration, glial cell activation, and inflammation. In addition, The SiO_2_ NPs caused oxidative stress, as demonstrated by the increase in the ROS indicator H_2_DCF-DA. Furthermore, the pretreatment of R28 cells with N-acetylcysteine, an ROS scavenger, attenuated the ROS production and cytotoxicity induced by SiO_2_ NPs.

**Conclusions:**

These results provide evidence that SiO_2_ NPs induce size-dependent retinal toxicity and suggest that glial cell activation and ROS generation contribute to this toxicity.

**Graphical Abstract:**

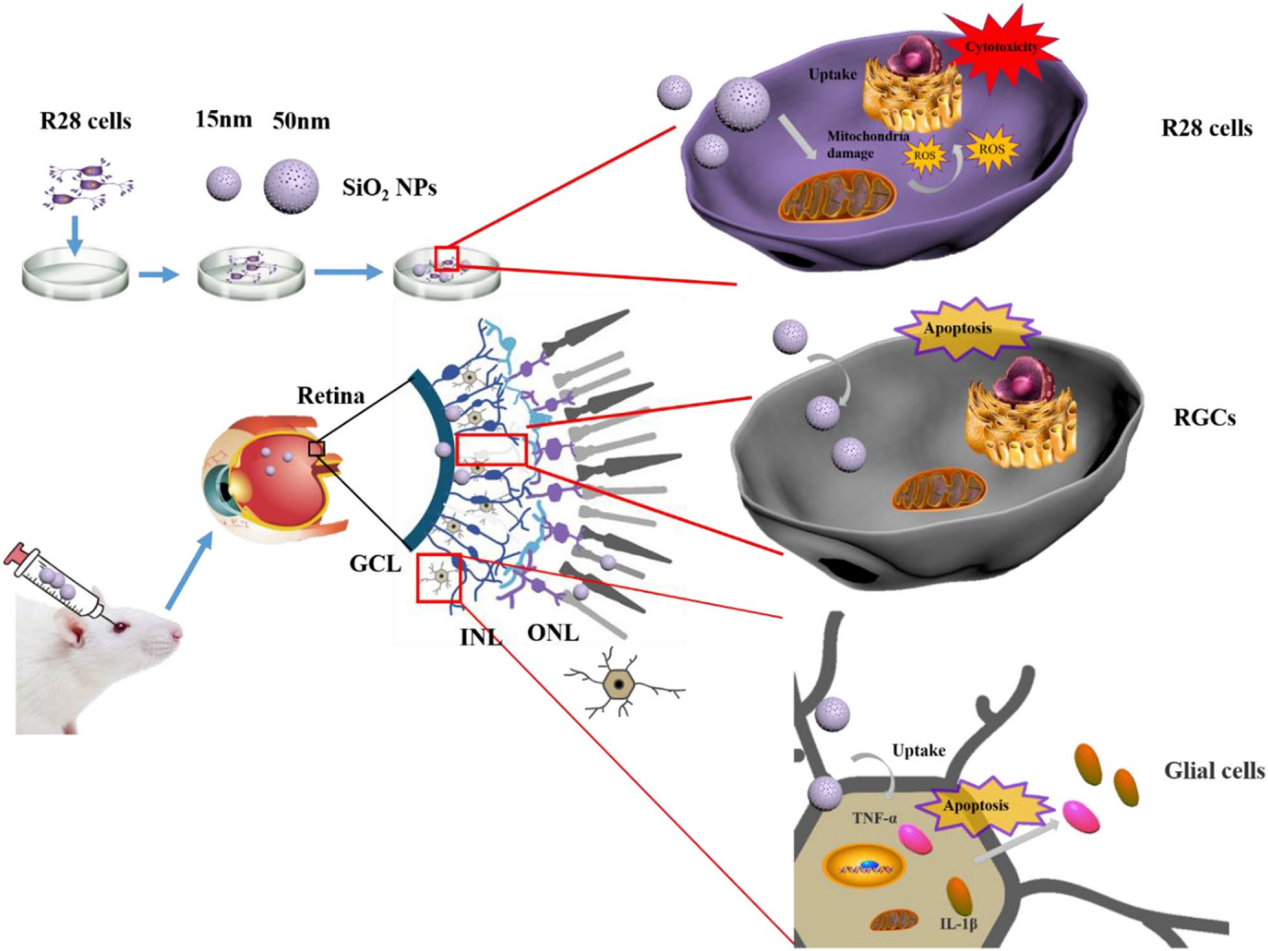

**Supplementary Information:**

The online version contains supplementary material available at 10.1186/s12951-022-01326-8.

## Introduction

Due to the wide application of nanomaterials in various fields, they are being increasingly manufactured. Silica nanoparticles (SiO_2_ NPs) are abundant in the Earth, and over 100 SiO_2_ NP products have been released on the global market [1–3]. In the last decade, SiO_2_ NPs have shown promise for applications in the biomedical field, including in disease labeling, biosensors, and the delivery of drugs and vaccines, due to their thermal stability and biocompatibility [4, 5]. Mesoporous and core/shell SiO_2_ NPs have been developed for tumor imaging and therapy [6]. In addition, SiO_2_ NPs have been reported for application in the treatment of ocular diseases. For example, SiO_2_ NPs loaded with a nitric oxide donor can be used to treat primary open-angle glaucoma [7]. The intravitreal injection of SiO_2_ NPs was shown to significantly inhibit retinal angiogenesis in oxygen-induced retinopathy mice [8]. However, the growing potential for human exposure to SiO_2_ NPs has attracted concern surrounding human health.

Some in vivo and in vitro studies have shown that SiO_2_ NPs can cause toxicity to different organs in the human body; toxicity has been demonstrated in lung epithelial cells [9], liver cells [10], intestinal cells [11], the lungs [12], and kidneys [13]. In addition, SiO_2_ NPs were found to induce genotoxicity and alterations in gene and protein expression [12, 14], and Chen et al. [15] reported that SiO_2_ NPs can induce cornea toxicity. The local ocular delivery of medicine has become an important strategy for treating retinal diseases [16–18], and intravitreal injection has become a common treatment method for various retinal diseases including diabetic retinopathy, macular degeneration, macular edema, and inflammatory diseases [19, 20]. However, the use of intravitreal drugs can result in retinal toxicity [21]. For example, silver nanoparticles were found to induce apoptosis in the human retinal pigment epithelia cell line ARPE-19 in vitro [22]. Wang et al. [23] reported that ZnO nanoparticles induced murine photoreceptor cell death in vitro. Nanomaterials can also induce ocular inflammation [24], and inflammation of the retinal tissue can lead to the secretion of cytokines and retinal damage. However, little is currently known about the retinal toxicity induced by SiO_2_ NPs in vitro and in vivo.

The effects of nanomaterials on cells may be influenced by various properties, including the nanomaterial’s crystallinity, size, shape, and surface area. Natural silica can exist in two states: crystalline and amorphous. Crystalline silica can easily lead to silicosis and lung cancer and is classified as a Group 1 carcinogen by the International Agency for Research on Cancer (IARC) [1, 25]. Notably, the toxicity of amorphous SiO_2_ NPs has begun to attract the attention of scientists in recent years. Mice treated with 70-, 300-, and 1000-nm amorphous SiO_2_ NPs showed no changes in hematology, histopathology, or biochemistry [26]; in contrast, Liu et al. [1] found that amorphous SiO_2_ NPs induced inflammation in HUVEC cells by activating the HMGB1/TLR4/MYD88/NF-kb signaling pathway. Tassinari et al. [27] that amorphous SiO_2_ NPs induced acute toxicity in the liver and spleen of male and female rats after intravenous administration. Brandão et al. [12] demonstrated that amorphous SiO_2_ NPs induced genotoxicity in lung cells. Particle size is also an important factor affecting the toxicity of SiO_2_ NPs. For example, in the A549 cell line, SiO_2_ NPs induced higher toxicity than SiO_2_ microparticles [28]. An in vitro study showed that SiO_2_ NP-induced toxicity depends not only on the particle size, but also on the cell type [29]. In the present study, we investigated the effect of amorphous SiO_2_ NP size on retinal toxicity.

The mechanism of SiO_2_ NP-induced toxicity remains unclear. One potential mechanism by which SiO_2_ NPs might induce retinal toxicity involves reactive oxygen species (ROS). Some reports have suggested that SiO_2_ NPs can induce ROS production in various cell lines, including human keratinocytes [30], human HK-2 cells [31], and HUVEC cells [1]. Many studies have indicated that ROS are closely related to cytotoxicity [32, 33], and the inhibition of ROS can ameliorate the cytotoxicity. Other studies have demonstrated that SiO_2_ NPs can induce inflammation [11], and oxidative stress and inflammation are thought to be closely related to the toxicity induced by nanoparticles.

Previous studies generally focused on a limited number of endpoints; in contrast, in the current study, we implemented a comprehensive set of tests to assess the potential effects of SiO_2_ NPs on the retina and retinal cells. More specifically, the cytotoxicity, morphological changes, and localization of SiO_2_ NPs in R28 cells induced by SiO_2_ NPs with two different sizes (15 and 50 nm) were investigated. We also determined the role of SiO_2_ NPs in the apoptosis of retinal cells and the damage of retinal ganglion cells (RGCs). In addition, we explored the potential role of glial cell activation, inflammation and ROS accumulation in SiO_2_ NP-induced retinal toxicity.

## Materials and methods

### Chemicals and reagents

SiO_2_ NPs were purchased from Shanghai Macklin Biochemical Co., Ltd (Shanghai, China). Fetal bovine serum (FBS), Dulbecco’s modified eagle medium (DMEM), and penicillin/streptomycin were obtained from Life Technologies (Carlsbad, CA, USA). N-acetylcysteine (NAC) and 2′,7′-dichlorofluorescin diacetate (H_2_DCF-DA) were purchased from Sigma-Aldrich (St. Louis, MO, USA). A JC-1 mitochondrial membrane potential assay kit was purchased from Beyotime Biotechnology Co., Ltd. (Shanghai, China).

### Characterization of SiO_2_ NPs

The sizes and morphologies of the SiO_2_ NPs were evaluated by transmission electron microscopy (TEM; Tecnai F20, Philips, Netherlands, 200 kV) and field-emission scanning electron microscopy (FE-SEM; JEOL JSM-7001F). The crystal structures of the SiO_2_ NPs were examined by powder X-ray diffraction (XRD) using monochromic Cu-Kα radiation (Rigaku Smart Lab, Japanese Neo Confucianism, Japan) at 40 kV and 300 mA. To investigate the chemical states of the SiO_2_ NPs an X-ray photoelectron spectrophotometer (XPS) was employed. XPS analyses of the nanoparticles were conducted by Axis Ultra DLD instrument (Kratos Analytical, Manchester, UK).

### Size and stability characterization of SiO_2_ NPs in cell culture medium

Due to concerns about the interaction between medium components, serum and SiO_2_, the stability of SiO_2_ NPs in cell culture media was investigated. SiO_2_ NPs were suspended at a concentration of 100 ug/ml in DMEM media with 10%, 1% or without FBS, and ultrapure water for 12 and 24 h in a humidified incubator prior to Hydrodynamic diameter and Zeta (ζ) potential analysis, which were measured using Zetasizer Nano (Malvern, Worcestershire, UK).

### Cell culture and treatment with SiO_2_ NPs

The retinal precursor cell line R28 was obtained from Kerafast (Boston, MA, USA) and cultured according to the supplier’s instructions. The cells were cultured in DMEM+ , which contained 420 mL DMEM (Sigma-Aldrich, St. Louis, MO, USA), 15 mL sodium bicarbonate (Sigma-Aldrich, St. Louis, MO, USA), 50 mL calf serum (Hylone), 5 mL MEM non-essential amino acids (GIBCO), 5 mL L-glutamine (GIBCO), and 0.625 mL Gentamicin (80 mg/mL; Solarbio Life Sciences Co., Ltd, Beijing, China). The cells were maintained at 37 °C in a humidified atmosphere containing 5% CO_2_. The human retinal pigment epithelial cell line (ARPE-19 cells) was purchased from the Fu Heng Cell Center (Shanghai, China). The cells were cultured in DMEM/F-12 medium (GIBCO) containing 10% FBS, penicillin (50 U/mL), and streptomycin (50 U/mL). Cells were cultured at 37 °C in a humidified atmosphere with 5% CO_2_. SiO_2_ NPs were dispersed in ultrapure water to prepare a stock solution (200 mg/mL). The stock solution was sonicated using a probe sonicator (Ningbo Xinzhi Biotechnology Co., Ltd., Ningbo, China) at 600 W for 40 min (pulse on: 2 s and pulse off: 2 s in an ice-bath) and diluted to different concentrations with culture medium just before cell exposure. The cells were adjusted to a concentration of 1 × 10^5^ cells/mL in a volume of 100 μL per well in 96-well plates for toxicity assays. NP suspensions were freshly prepared before the treatments, and diluted to various concentrations with the FBS-free culture medium, then immediately applied to the cells.

### Measurement of cellular ATP levels and lactate dehydrogenase (LDH) release

Cell Titer-Glo ® Luminescent Cell Viability Assay (Promega, Madison, WI, USA) was used to monitor the ATP levels in SiO_2_ NP-treated ARPE-19 or R28 cells according to the manufacturer’s instructions. Briefly, cells were cultured in 96-well plates and hatched overnight. After the cells were adhered, 15 and 50-nm SiO_2_ NPs were added in a dose-dependent manner (0, 5, 10, 20, 40, and 80 μg/mL) into the culture medium and cultured for 12 or 24 h. After washed off the culture medium with PBS, 100 μL of Cell Titer-Glo reagent was added per well. Cells were incubated at 37 °C in 5% CO_2_ for 10 min, and luminescence was recorded using a Synergy H4 Hybrid microplate reader (BioTek, Winooski, USA). Cellular ATP levels were calculated by comparing the luminescence intensity of the treated cells to that of the vehicle control. The cytotoxicity of SiO_2_ NPs was assessed using the LDH Release Assay (Beyotime, Beijing, China) as our previous method [34]. The absorbance was detected at a wavelength of 490 nm by using a Synergy H4 Hybrid microplate reader and data are presented as relative values compared to control.

### Cell morphology

R28 cells were seeded in 96-well plates at a density of 1 × 10^4^ cells/well and cultured overnight in a CO_2_ incubator. The cells were exposed to SiO_2_ NPs at different concentrations (5–80 µg/mL) for 12 and 24 h. The changes in cell morphology were examined using a phase-contrast microscope (Leica DM16000B, Heidelberg, Germany).

### Uptake of SiO_2_ NPs

To examine the localization of SiO_2_ NPs in R28 cells, R28 cells were plated in six-well plates, cultured overnight, and treated with SiO_2_ NPs (20 µg/mL) for 24-h. The R28 cells were then collected, washed three times with phosphate-buffered saline (PBS), and fixed with 2.5% glutaraldehyde solution at 4 °C overnight. After the fixed cells were dehydrated, serial ultrathin sections were created and examined by TEM (Hitachi H7650, Japan).

### Detection of mitochondrial membrane potential

The SiO_2_ NP-induced changes in mitochondrial membrane potential were assessed as previously described [34]. Briefly, R28 cells were seeded in dishes at a density of 1 × 10^5^ cells/mL and cultured overnight. The cells we treated with different concentrations of SiO_2_ NPs for 6, 12 and 24 h. Following treatment, the cells were washed three times with PBS and then incubated with JC-1 (20 μM; Beyotime, Beijing, China) for 15 min. After removing the JC-1 staining solution, the cells were washed three times, and PBS was added for imaging by confocal laser scanning microscopy (CLSM; ZEISS LSM 800, Germany).

### Intravitreal injection of SiO_2_

Three-week-old male Sprague–Dawley rats were purchased from Pengyue Experimental Animal Company (Jinan, China). All animal procedures were carried out in accordance with the National Institutes of Health Guide for the Care and Use of Laboratory Animals and were in compliance with the ARVO Statement for the Use of Animals in Ophthalmic and Vision Research. Animal protocols were approved by the Committee of Yantai University for the Care and Use of Laboratory Animals. All rats were housed under 12-h dark/light cycles at 23 °C ± 1 °C, and food and water were available ad libitum. Prior to intravitreal injection, the rats were anesthetized via the intraperitoneal injection of ketamine (100 mg/kg) and xylazine (7 mg/kg), and the pupils were anesthetized with 0.5% proxymetacaine hydrochloride. The rats were randomly divided into three groups: sham, vehicle control (PBS), and SiO_2_ NPs. Intravitreal injections were carried out using a 30-gauge needle attached to a 1-mL syringe; 5 μL of SiO_2_ NP suspension was injected to obtain a final concentration of 80 μg/mL in the vitreous humor.

### TUNEL assay

Assays were performed using a one-step TUNEL Apoptosis Assay Kit (Beyotime) following the manufacturer’s instructions. Briefly, cryosections were stained using the kit to test DNA fragmentation as an indicator of cell death. To count TUNEL-positive cells, three sections from each rat were imaged by confocal laser scanning microscopy (CLSM; ZEISS LSM 800, Germany). The counts from all sections of the same animal were averaged, and the data from six rats were used to obtain the average and standard deviation (SD) for the group.

### Evaluation of retinal ganglion cells (RGCs) and inflammatory markers in SiO_2_ NP-injected retinas by immunofluorescence

Immunostaining was performed following the previously described method. Briefly, cryosections were permeabilized with 0.5% Triton X-100 in PBS for 15 min, blocked with 5% bovine serum albumin (BSA) for 1 h at room temperature, and then stained with primary antibodies in blocking solution overnight at 4 °C prior to incubation with secondary antibodies diluted in blocking solution for 1 h at room temperature. Nuclei were stained with 4′,6-diamidino-2-phenylindole (DAPI). The primary antibodies were as follows: mouse anti-β-III-tubulin (1:100, Beyotime), rabbit anti-glial fibrillary acid protein (anti-GFAP; 1:100, Beyotime), rabbit anti-TNF-α (1:100, Beyotime), and IL-1β (1:100, Beyotime). To quantify immunofluorescence intensity, the areas of β-III-tubulin and GFAP immunopositivity were determined by thresholding based on the images obtained using Image J (National Institutes of Health, Bethesda, MD, USA). TNF-α- and IL-1β-positive cells were quantified from at least three sections of each rat. Each group included six rats. Comparisons between groups were made using one-way analysis of variance (ANOVA).

### Measurement of ROS

Intracellular ROS production was assessed using H_2_DCF-DA staining as previously described [34]. Briefly, R28 cells were treated with 10 μM H_2_DCF-DA for 30 min in the cell culture incubator. The cells were washed with PBS to remove unincorporated dye and then treated with 5–80 μg/mL SiO_2_ NPs in phenol red-free medium. The cells were incubated for 24 h, and the fluorescence intensity was measured after 2, 4, 6, 12, and 24 h using a Synergy H4 Hybrid microplate reader. Meanwhile, the oxidation of H_2_DCF-DA was detected by CLSM (ZEISS LSM 800, Germany) at the same time points.

### Statistical analysis

All data are presented as the mean ± SD. Statistical analysis was performed using Graph Pad Prism 6 (La Jolla, CA, USA). Treatment-related differences were evaluated by one-way ANOVA followed by Dunnett’s tests (for comparisons of different concentrations to the vehicle control) or by two-way ANOVA followed by Tukey’s multiple comparison test (for comparisons of two treatment groups in NAC pretreatment experiments). A difference was considered statistically significant when the *p* value was less than 0.05.

## Results

### Characterization of SiO_2_ NPs

The wide application of SiO_2_ NPs in the biomedical field has raised concerns regarding the safety of these NPs in humans and the environment. While the cytotoxicity of SiO_2_ NPs has been investigated by numerous scientists [3, 5, 35], most of these studies explored various SiO_2_ NP characteristics using a wide variety of in vitro models. Until now, limited studies have evaluated the retinal toxicity both in vitro and in vivo. In the present study, we explored the retinal toxicity of two types of SiO_2_ NPs with different sizes both in vitro and in vivo. We also investigated the potential mechanism underlying the retinal toxicity induced by SiO_2_ NPs.

Recently, SiO_2_ NPs have shown great potential in the treatment of ocular diseases [36–39]. Given the widespread use of SiO_2_ NPs to treat ocular diseases, the ocular toxicity of SiO_2_ NPs requires more attention from scientists and ophthalmologists. Park et al. reported that SiO_2_ NPs with sizes of 50, 100, and 150 nm did not induce significant cytotoxicity in cultured human corneal epithelial cells [40]. However, Chen et al. reported that SiO_2_ NPs led to cytotoxicity, ROS generation, and DNA damage in the human cornea [15]. SiO_2_ NPs can be used as intravitreal drug carriers [38, 41]; however, to the best of our knowledge, the retinal toxicity of SiO_2_ NPs has not been investigated before now. Therefore, we conducted both in vitro and in vivo experiments to evaluate the retinal toxicity of SiO_2_ NPs with sizes of 15 and 50 nm. The in vitro study used human R28 retinal precursor cells, which are expected to mimic in vivo responses.

**Fig. 1:**
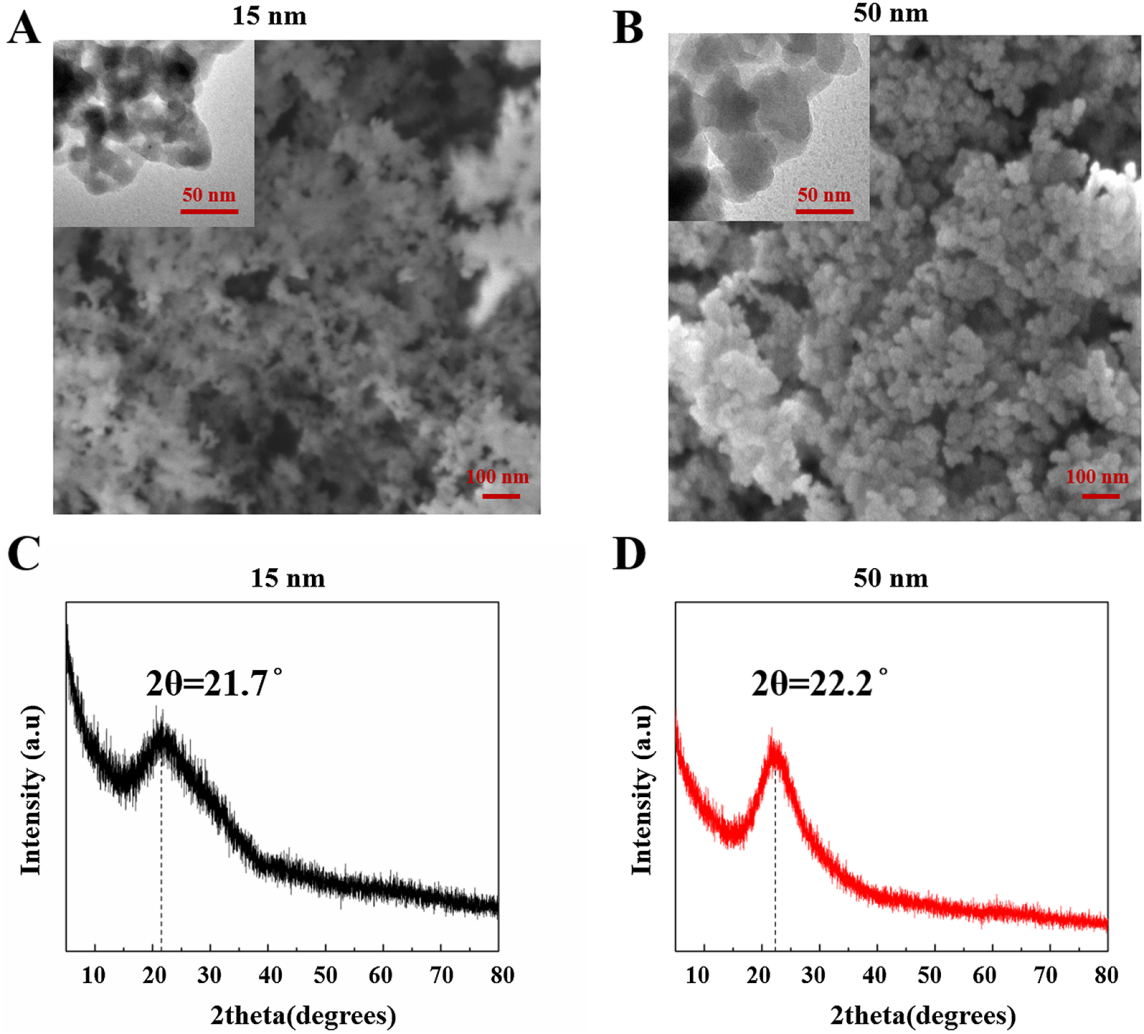
Characterization of SiO_2_ NPs. SEM and TEM (insets) images of (**A**) 15-nm SiO_2_ NPs and **B** 50-nm SiO_2_ NPs. XRD spectra of 15-nm SiO_2_ NPs (**C**) and 50-nm SiO_2_ NPs (**D**)

We first evaluated the effect of SiO_2_ NP size on retinal toxicity. The morphology, size, and structure the of SiO_2_ NPs were characterized by SEM, TEM, and XRD, respectively. The SEM images of the two types of SiO_2_ NPs (Fig. 1A and B) indicate that both NPs had spherical morphologies. The TEM images (insets of Fig. 1A and B) show that the SiO_2_ NPs had sizes of approximately 15 and 50 nm and were slightly aggregated in aqueous solution. As shown in Fig. 1C and D, the XRD patterns confirmed the amorphous nature of the 15 and 50 nm SiO_2_ NPs. There is no Bragg peak observed. The diffraction pattern of SiO_2_ NPs exhibited a single broad peak centered at ~ 22°, which is the characteristic peak for amorphous SiO_2_ NPs.

**Fig. 2:**
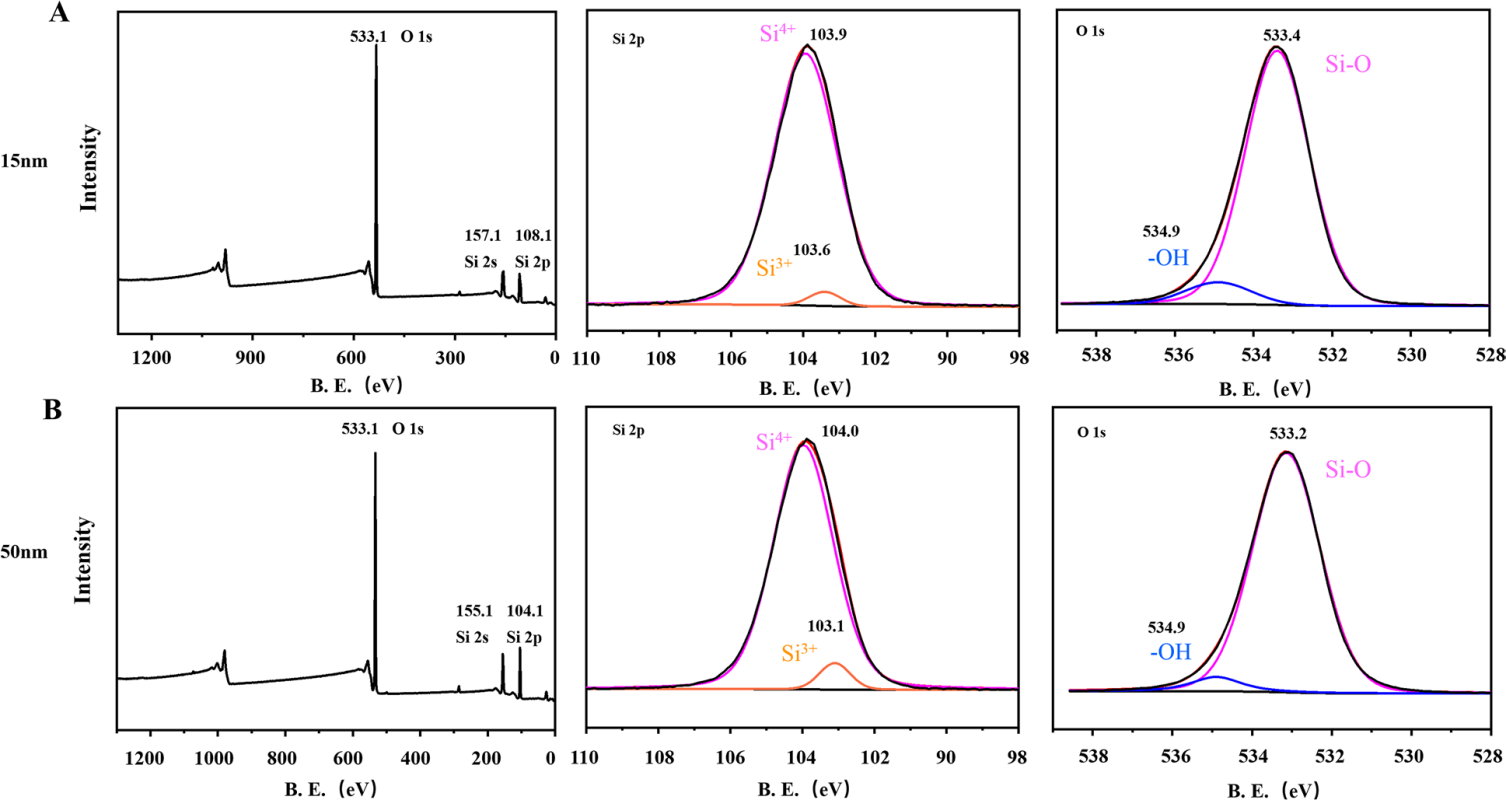
X-ray photoelectron spectroscopy SiO_2_ NPs. The main element composition in SiO_2_ NPs and valence state of Si atoms are shown in **A** (15 nm) and **B** (50 nm)

In order to investigate the chemical state of the elements of SiO_2_ NPs, XPS was performed. The wide-scan XPS survey spectrum of the NPs validates the existence of Si and O atoms, consistent with other studies. The high-resolution XPS spectrum of Si2p for both samples displayed two peaks, which indicated the main peak presented as Si (IV), and the other peak was matched well with Si (III), which was only a small mount. These results were consistent with other studies (see, for example, ref [42]). The O (1 s) spectra for both samples showed two peaks (Fig. 2). The main peak around 533eV was matched to the O^2-^ of SiO_2_, and the other peak at 534.9 eV can be fitted to the weakly bonded oxygen of water molecules adsorbed on the surface of NPs and possibly the OH functional group of SiO_2_, which is similar to other reports [43, 44]

To investigate about the aggregation of the SiO_2_ NPs by FBS and cell culture medium, the Hydrodynamic size and Zeta-Potential of SiO_2_ NPs in different suspensions (ultrapure water, DMEM, DMED + 1%FBS and DMEM + 10%FBS) were measured at 12 h and 24 h of incubation. As shown in Table 1, both 15- and 50-nm SiO_2_ NPs aggregated to various degrees in different suspensions. NPs slightly aggregated in water and DMEM suspensions. It is obvious that FBS can promote the aggregation of NPs more than DMEM. And, this effect was dose-dependent. Especially when the FBS content was increased to 10%, the phenomenon became more prominent, and the hydrodynamic size of both NPs in the medium + 10% FBS suspension was 2–3 times larger than that in water. In contrast, the hydrodynamic size of the FBS-free DMEM suspension is very similar to that of the water suspension. Consistent with this, changes in ζ potential also confirmed the aggregation phenomena (Table 2). Assessing the surface charge and stability of NPs are two widely uses of ζ potential [45]. Table 2 showed the ζ potential measurements of 15- and 50-nm SiO_2_ NPs in different kinds of suspension. The ζ potential values of all samples ranged from − 6 mV to − 20 mV. This is similar with one study [46], which the potential values were negative. Our own results showed that the potential values of ultrapure water and FBS-free DMEM ranged from − 10 mV to − 20 mV, which can be considered as a relatively stable state [45]. However, ζ potential values of DMEM + 10% suspension changed to a highly unstable state (0 ~ −10 mV) [47]. Notably, in our study, this aggregation was not time-dependent. Hence, to avoid the effect of this aggregation induced by FBS on toxicity, in the following in vitro assays, we used the FBS-free medium to disperse the NPs and sonicated for 40 min in ice-bath before all the treatments.

**Table 1:** The Hydrodynamic Diameter of SiO_2_ NPs in different suspensions

Size (nm)	Time (hour)	Utrapure water	DMEM	DMEM + 1% FBS	DMEM + 10% FBS (nm)^a^
15	12	140.3 ± 7.5	140.6 ± 4.8	178.9 ± 10.9	364.6 ± 14.1
	24	153.7 ± 31.3	150.6 ± 6.7	165.6 ± 3.6	381.7 ± 7.6
50	12	239.0 ± 1.7	254.8 ± 4.0	260.4 ± 4.0	381.3 ± 24.7
	24	252.8 ± 22.8	260.7 ± 8.9	243.0 ± 6.4	434.4 ± 12.5

^a^Data are expressed as mean ± standard deviation (SD)

**Table 2:** The Hydrodynamic Diameter of SiO_2_ NPs in different suspensions

Size (nm)	Time (hour)	Utrapure water	DMEM	DMEM + 1% FBS	DMEM + 10% FBS (nm)^a^
15	12	− 19.4 ± 1.2	− 12.6 ± 1.2	− 16.4 ± 0.8	− 6.8 ± 0.3
	24	− 17.4 ± 0.1	− 13.6 ± 1.7	− 17.6 ± 1.5	− 6.7 ± 1.0
50	12	− 15.7 ± 0.2	− 11.8 ± 1.7	− 10.0 ± 0.5	− 6.6 ± 1.1
	24	− 13.3 ± 0.7	− 13.0 ± 1.1	− 9.2 ± 2.6	− 6.7 ± 0.8

^a^Data are expressed as mean ± standard deviation (SD)

### Cytotoxicity of SiO_2_ NPs in R28 and ARPE-19 cells

**Fig. 3:**
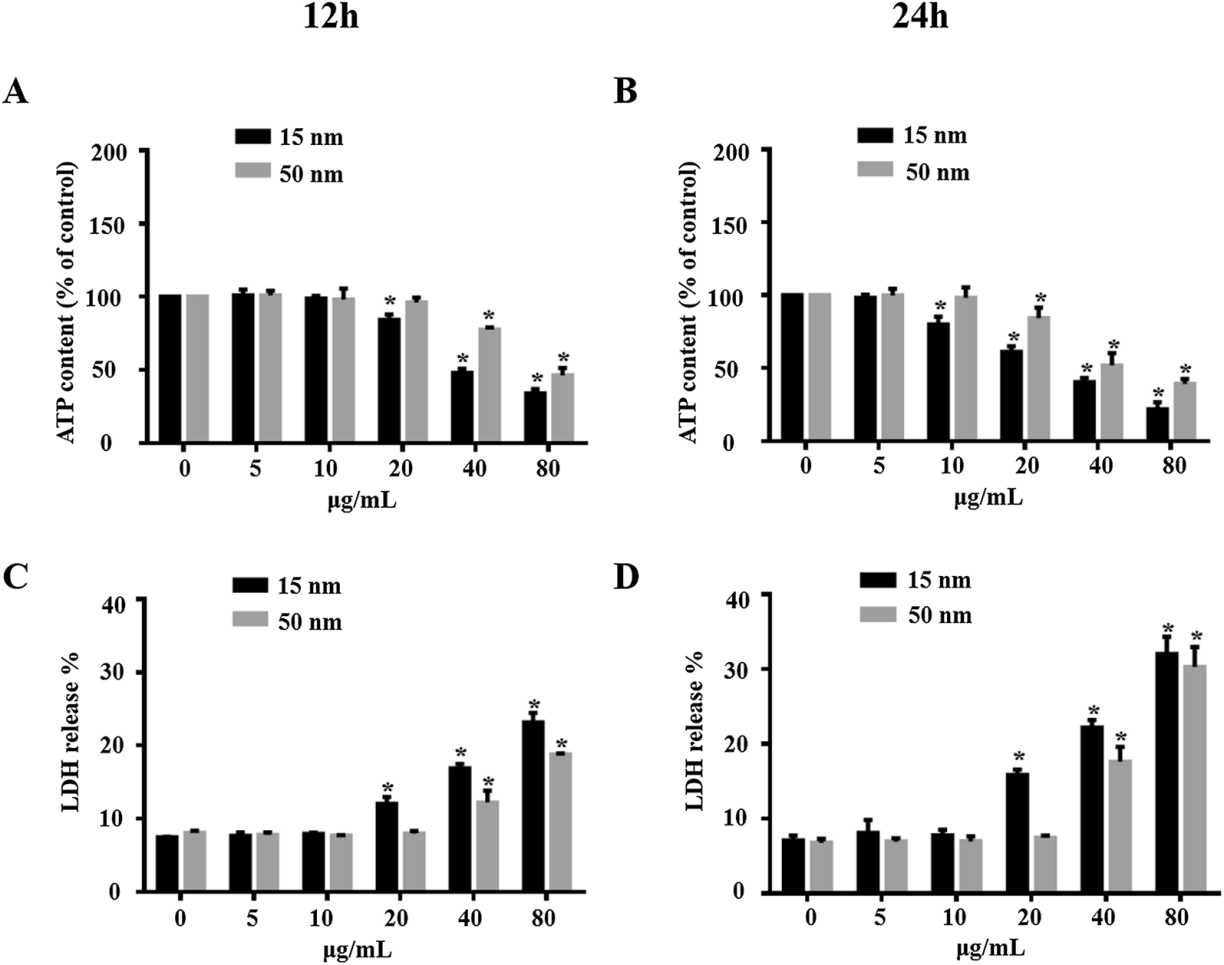
SiO_2_ NPs induce cytotoxicity in R28 cells. R28 cells were exposed to different concentrations (5–80 μg/mL) of SiO_2_ NPs for (**A** and **C**) 12 h and (**B** and **D**) 24 h before measurements of (**A** and **B**) ATP content and (**C** and **D**) LDH release. Data points represent the mean ± SD from three independent experiments with three samples per concentration in each experiment. *p < 0.05 compared to the control

We compared the cytotoxicity of the SiO_2_ NPs with different sizes (15 and 50 nm) in two cell lines, which are human R28 retinal precursor cells and ARPE-19 human retinal pigment epithelial cells. Cytotoxicity was determined by adenosine triphosphate (ATP) assay and lactate dehydrogenase (LDH) release assay. The R28 and ARPE-19 cells were treated with the two types of SiO_2_ NPs at various concentrations ranging from 5–80 μg/mL for 12 and 24 h. SiO_2_ NPs are much more toxic to R28 than to ARPE-19 As shown in Fig. 3, the SiO_2_ NPs induced significant time- and concentration-dependent decreases in ATP content (Fig. 3A & B) and LDH release (Fig. 3C & D). Among the two types of SiO_2_ NPs, the R28 cells showed higher sensitivity to the 15-nm SiO_2_ NPs. Furthermore, SiO_2_ NPs are much more toxic to R28 cells than to ARPE-19 cells (Additional file 1: Figure S1). Quantitative analysis showed that exposure of R28 cells to 15-nm NPs at a concentration of 20 μg /mL for 12 h resulted in a significant decrease in ATP content. However, ARPE-19 cells were exposed to 15 nm nanoparticles at a concentration of 80 μg /ml for 12 h to induce a similar phenomenon. The results from LDH release assay further confirmed this. Thus, in the following studies, our in vitro assays were all performed in R28 cells.

### SiO_2_ NPs induce morphological changes in R28 cells

**Fig. 4:**
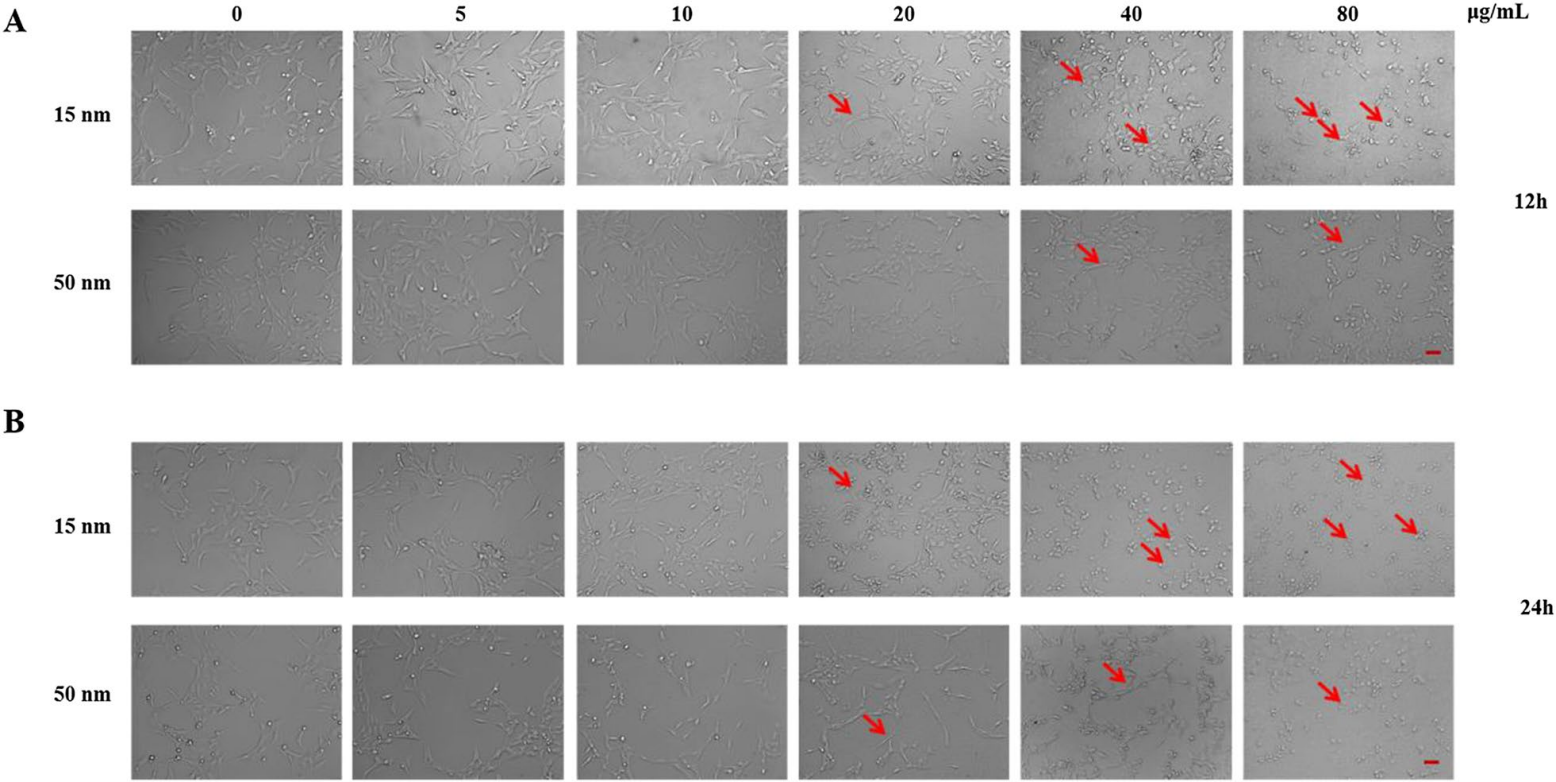
SiO_2_ NPs induce morphological changes in cells. Morphological changes were observed via microscopy in R28 cells after 12 h (**A**) and 24 h (**B**) of exposure to 15- and 50-nm SiO_2_ NPs at the indicated concentrations. Scale bar: 25 μm

The morphology of the R28 cells changed as the SiO_2_ NP concentration increased. After 12 h, the cell morphology became irregular when the NP concentration reached 20 (50-nm SiO_2_ NPs; Fig. 4A) or 40 μg/mL (50-nm SiO_2_ NPs; Fig. 4A). At 24 h, the changes in cell morphology became more prominent with increasing SiO_2_ NP concentration (Fig. 4B). At the concentration of 80 μg/mL, most cells were detached, and the density was obviously reduced.

### In vitro localization of SiO_2_ NPs in R28 cells

**Fig. 5:**
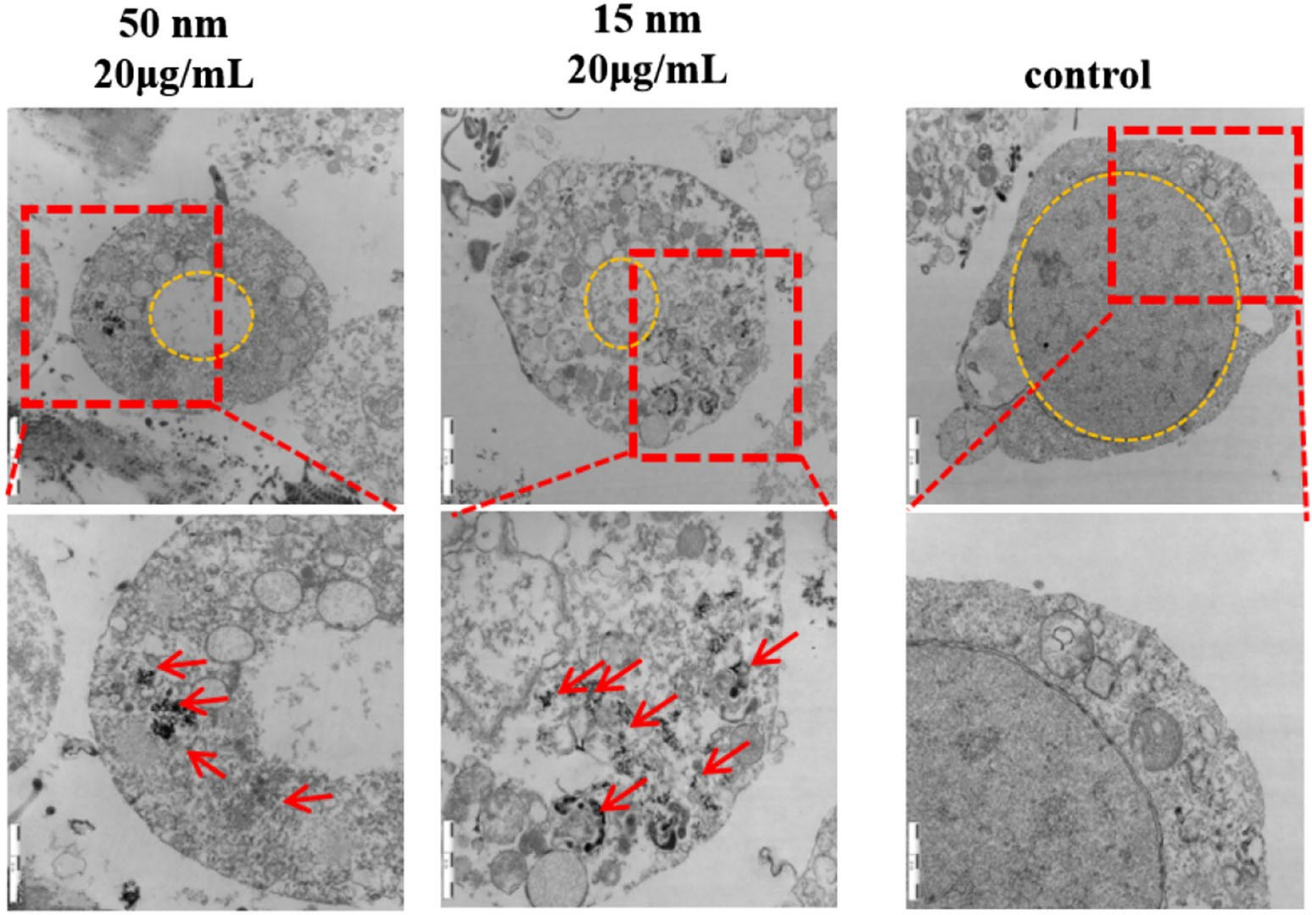
TEM evaluation of the cellular uptake and localization of 15- and 50-nm SiO_2_ NPs in R28 cells over 12 h. Scale bars: 1 μm and 2 μm

The in vitro distributions of SiO_2_ NPs with sizes of 15 and 50 nm in R28 cells were evaluated by TEM. In R28 cells before SiO_2_ NP treatment, no SiO_2_ NPs were observed in the nucleus or cytosol (red arrows) (Fig. 5). After exposure for 24 h, both 15- and 50-nm SiO_2_ NPs were visible in the cytoplasm, and some 15-nm SiO_2_ NPs were found in the mitochondria. Furthermore, nuclei were shrunken in cells treated with NPs.

### SiO_2_ NPs induce mitochondrial dysfunction

**Fig. 6:**
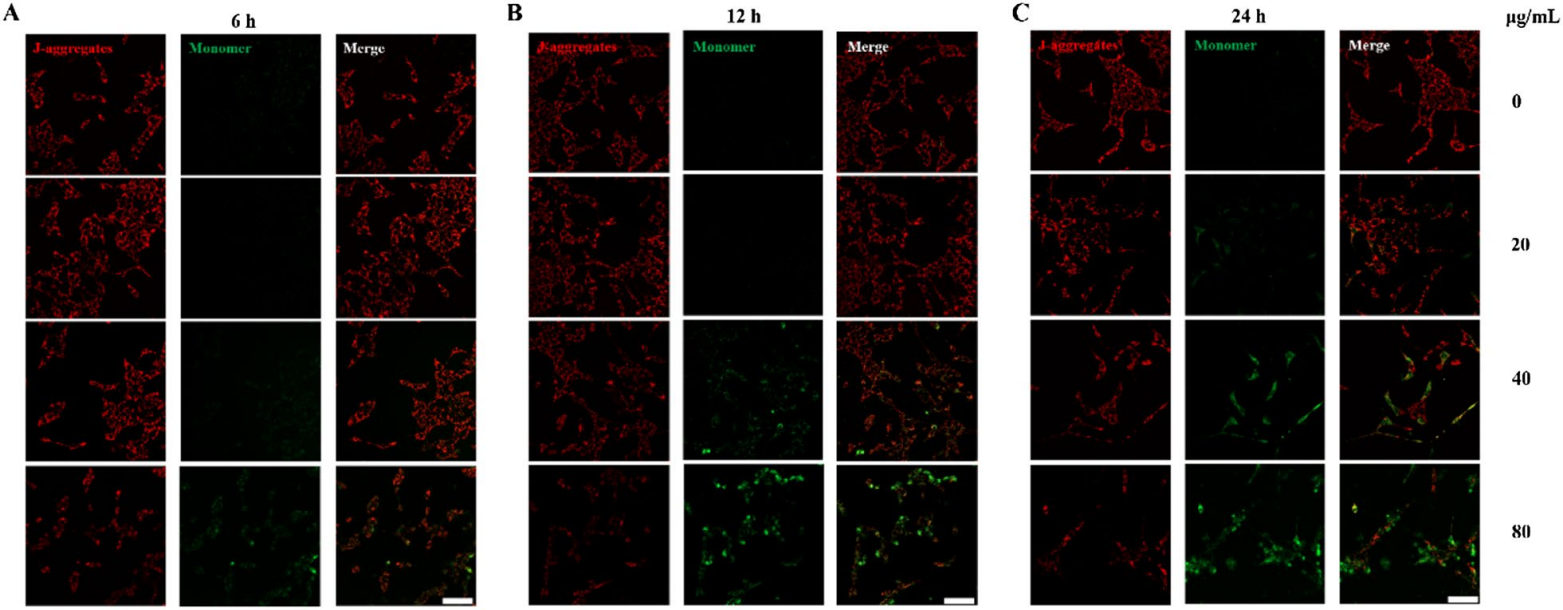
SiO_2_ NPs induce mitochondrial dysfunction. R28 cells were treated with three concentrations (20, 40, and 80 μg/mL) of SiO_2_ NPs for 6 h (**A**), 12 h (**B**) and 24 h (**C**), and mitochondrial membrane potential was evaluated by JC-1 staining. Scale bar: 50 μm

As the 15-nm SiO_2_ NPs accumulated in the mitochondria, we measured the change in mitochondrial depolarization (ΔΨm) in R28 cells treated with 15-nm SiO_2_ NPs. The value of ΔΨm was measured using JC-1 dye. The R28 cells were treated with SiO_2_ NPs at concentrations of 20, 40, and 80 μg/mL for 6, 12, and 24 h. Decreases in mitochondrial depolarization in the R28 cells were observed as early as 6 h after treatment with 80 μg/mL SiO_2_ NPs (Fig. 6A). As shown by the JC-1 staining images (Fig. 6C), the transition from red fluorescence to green fluorescence became more obvious at 24 h after treatment, suggesting that the SiO_2_ NPs induced a significant time- and concentration-dependent decrease in mitochondrial depolarization (Fig. 6). Consequently, in subsequent experiments, the SiO_2_ NP concentration of 80 μg/mL was used to investigate the retinal toxicity in vivo.

### SiO_2_ NPs induce retinal toxicity in vivo

**Fig. 7:**
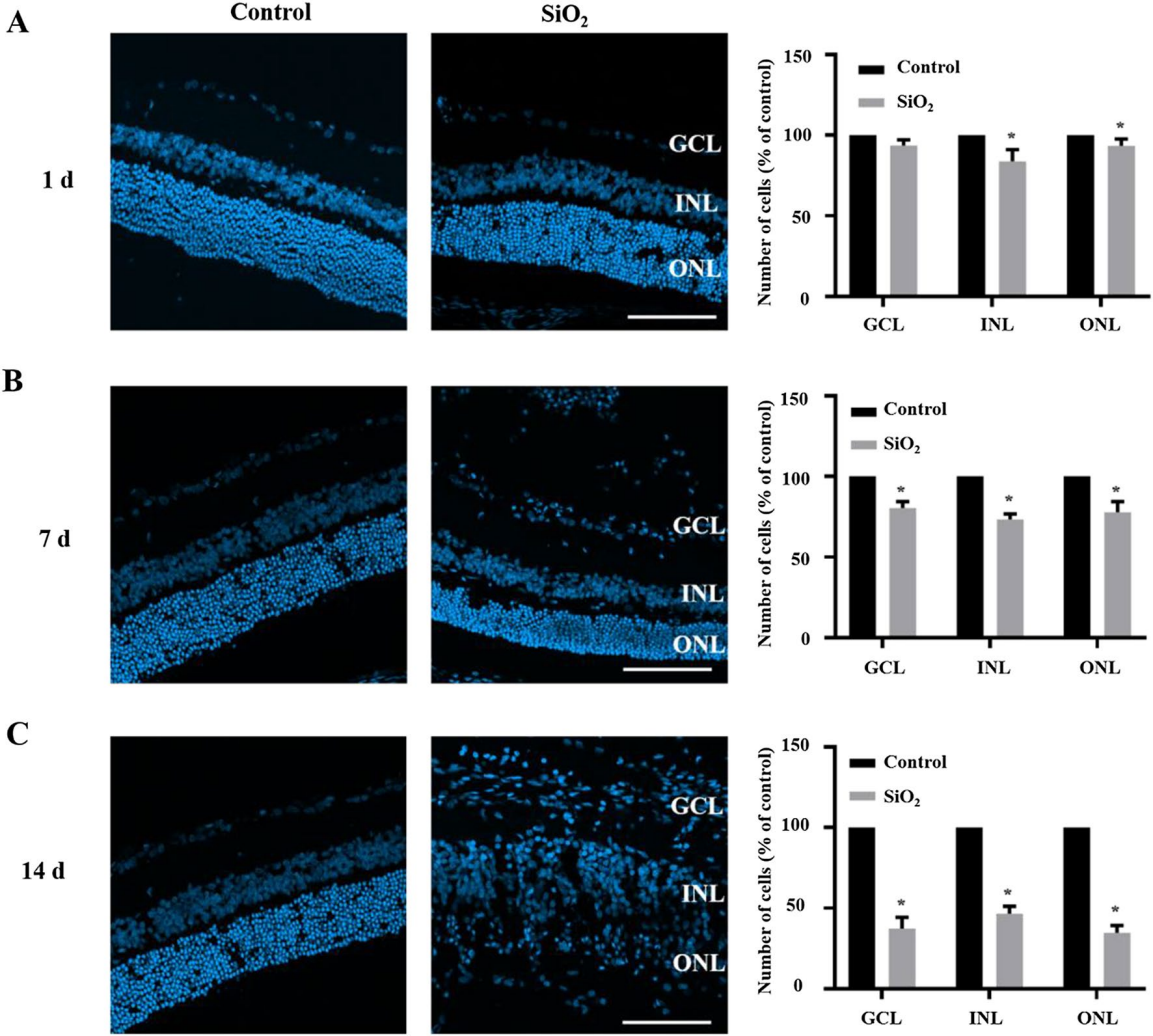
SiO_2_ NPs decrease the number of retinal cells. PBS or 15-nm SiO_2_ NPs were injected into the right eyes of rats. After euthanization, retinal sections were prepared for fluorescence microscopy. At 1 d (**A**), 7 d (**B**), and 14 d (**C**) after injection, the retinal layers (INL, GCL, and RGC layers) were stained by DAPI, and the cells in each layer were counted. Representative images are shown in the left panel, and the bar graph depicts the mean percentages of dead cells. *p < 0.05 compared to the vehicle control. Scale bar: 100 μm

**Fig. 8:**
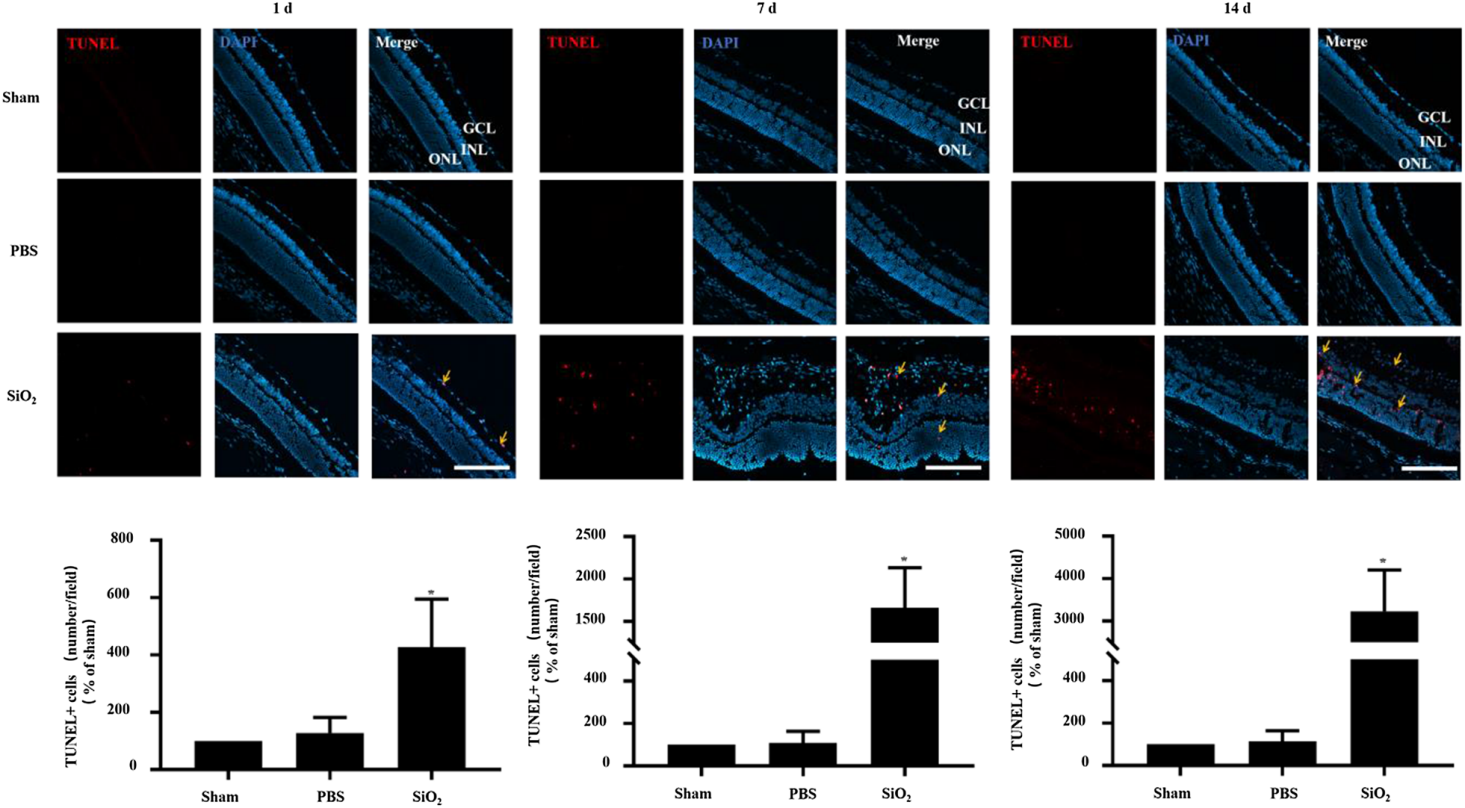
SiO_2_ NPs cause apoptosis in rat retinas. Retinal sections were prepared as described in Fig. 6. At 1 d (**A**), 7 d (**B**), and 14 d (**C**) after SiO_2_ NP injection, the retinas were analyzed by TUNEL assay (red = TUNEL; blue = DAPI). TUNEL-stained cells were observed in the GCL, INL, and ONL (orange arrows). Representative TUNEL staining images are shown in the upper panel. The bar graph depicts the mean percentages of apoptotic cells. *p < 0.05 compared to the sham group. Scale bar: 100 μm

To examine the retinal toxicity of SiO_2_ NPs in vivo, SiO_2_ NPs were intravitreally injected. At 1, 7, and 14 d after injection, the rats were euthanized, and frozen sections of the retina were prepared for fluorescence staining. The retina shape became irregular at 7 d after injection, and the retinas became very loose at 14 d after injection. Notably, many cells infiltrated into the retinal ganglion cell layer (GCL); these cells were suspected to be inflammatory cells. To measure retinal cell death after SiO_2_ NP injection, cells stained with DAPI were counted in the outer nuclear layer (ONL), inner nuclear layer (INL), and GCL. The number of cells decreased with time after injection in the ONL, INL, and GCL, and the overall number of cells also decreased (Fig. 7). Next, retinal cryosections were analyzed by TUNEL apoptosis assay (Fig. 8). The percentage of apoptotic cells increased in the SiO_2_ NP-treated groups in a time-dependent manner. Compared to the sham group, the intravitreal injection of SiO_2_ NPs increased the number of TUNEL-positive cells by approximately 4-, 16-, and 32-fold after 1, 7, and 14 d, respectively.

### SiO_2_ NPs activate the inflammatory response in vivo

**Fig. 9:**
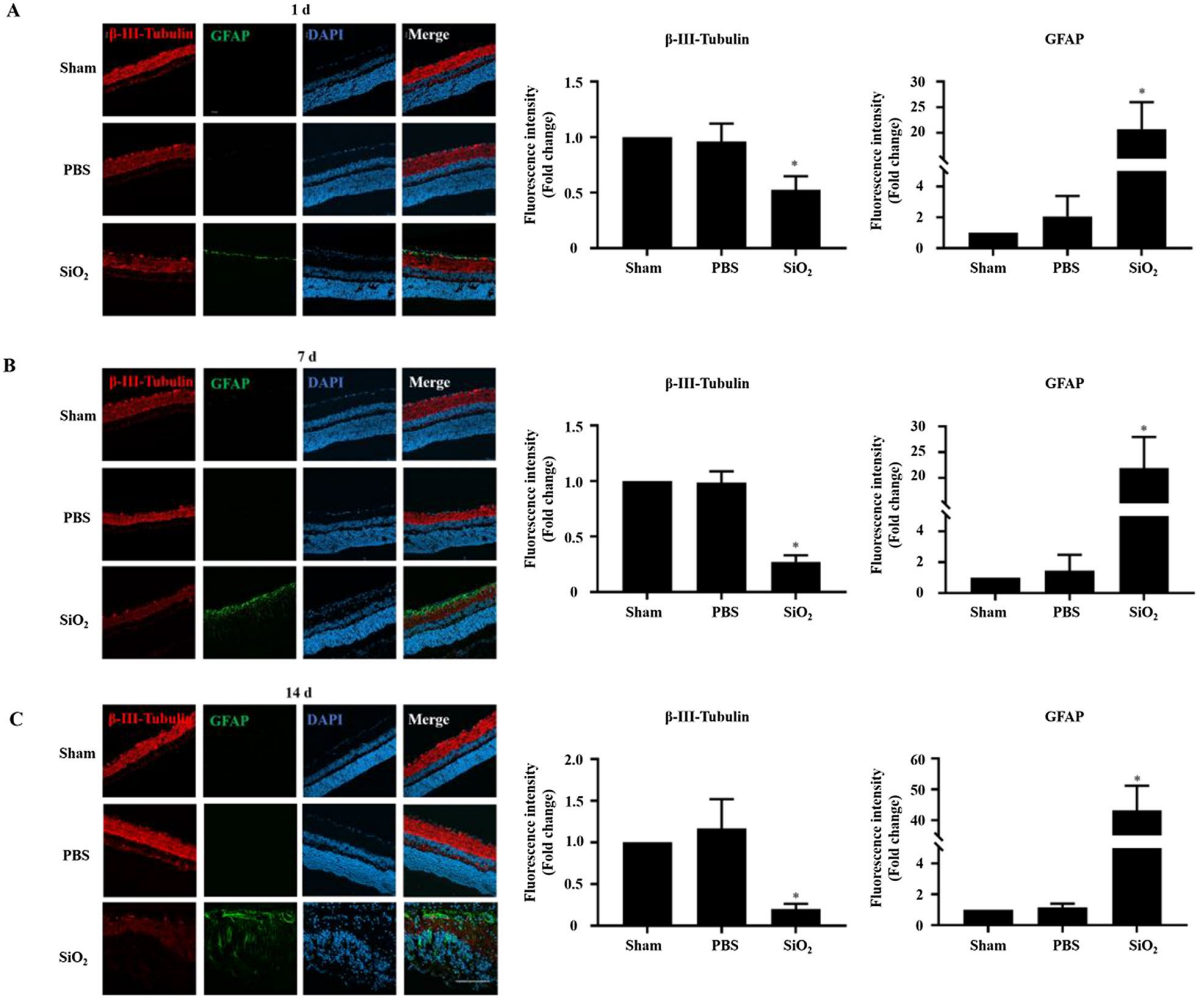
SiO_2_ NPs induce the activation of retinal glial cells and reduce RGCs. Retinal sections were prepared as described in Fig. 6. At 1 d (**A**), 7 d (**B**), and 14 d (**C**) after SiO_2_ NP injection, retinas were stained with the antibodies of β-III-tubulin (a marker of RGCs) and GFAP (a marker of glial cells). Representative images showing the distributions of β-III-tubulin (red) and GFAP (green). The bar graph depicts the mean fluorescence intensity. *p < 0.05 compared to the sham group. Scale bar: 100 μm

As mentioned above, cells that we suspected to be inflammatory cells infiltrated the GCL. Thus, we investigated whether the SiO_2_ NPs caused retinal inflammation. GFAP, a marker of glial cells in the retina, was assessed by immunofluorescence staining. As demonstrated in Fig. 9, glial cells were obviously activated as early as 1 d after the injection of SiO_2_ NPs, and the number of activated glial cells rose sharply at 7 d after injection (Fig. 9A and B). The maximum GFAP signal induction was approximately 60 times that of the PBS control at 14 d after injection (Fig. 9C). RGCs can be damaged by various stimuli such as inflammation, ischemia, oxidative stress, and excitotoxicity [48]. Therefore, to understand whether the activation of glial cells can damage RGCs, we evaluated the expression of β-III-tubulin, a marker of RGCs. As shown in Fig. 9A, at 1 d after SiO_2_ NP injection, the RGCs (β-III-tubulin positive) were reduced by 52% compared to the vehicle control. The cell number decreased more predominately at 7 and 14 d after injection (Fig. 9B & C). These findings demonstrate that the intravitreal injection of SiO_2_ NPs activated glial cells and damaged RGCs.

**Fig. 10:**
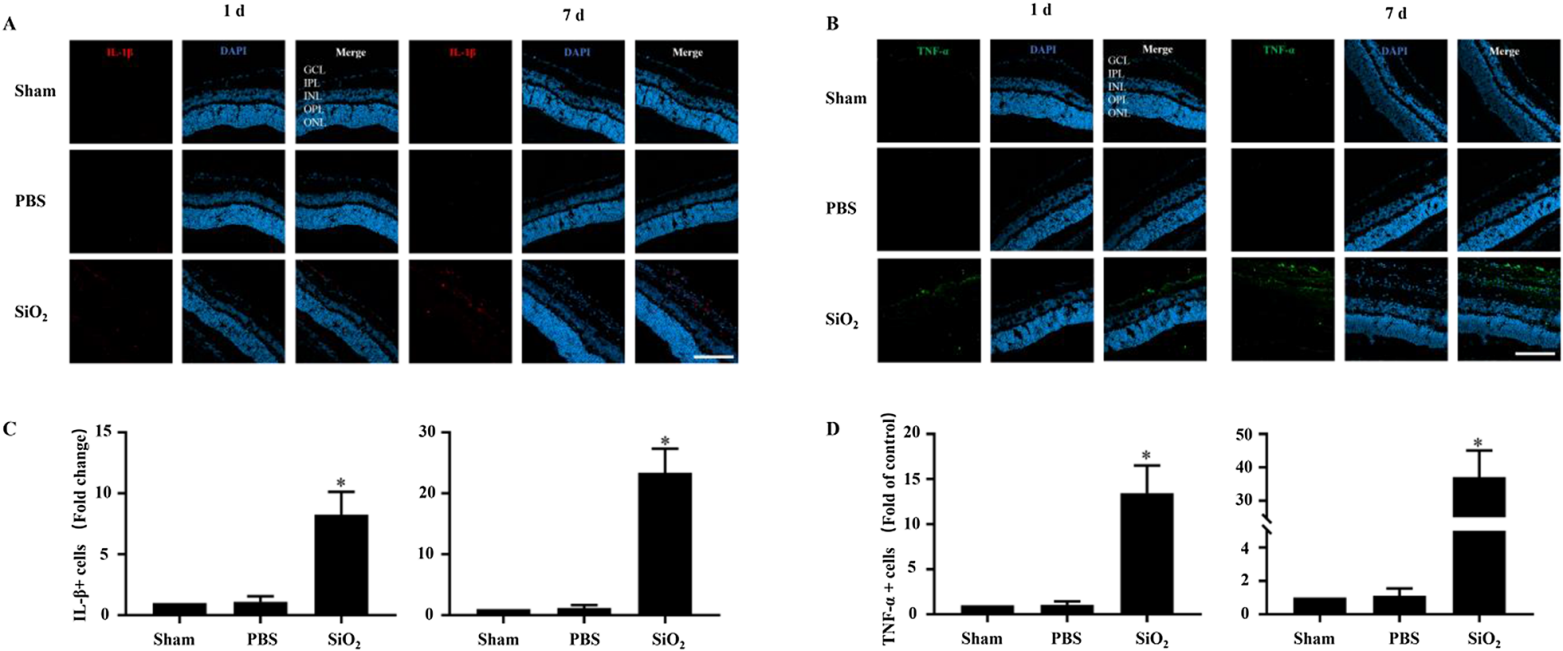
SiO_2_ NPs cause the secretion of IL-1β and TNF-α. Retinal sections were prepared as described in Fig. 7. At 1 d (**A** and **C**) and 7 d (**B** and **D**) after SiO_2_ NP injection, retinas were stained with the antibodies of TNF-α and IL-1β. Representative images showing the distributions of IL-1β (red) and TNF-α (green). The bar graph depicts the mean fluorescence intensity. *p < 0.05 compared to the sham group. Scale bar: 100 μm

As SiO_2_ NPs have been demonstrated to induce inflammation in HUVEC cells [1], and pro-inflammatory cytokines (e.g., TNF-α and IL-1β) secreted by macrophages play a crucial role in the inflammation process, we investigated whether the SiO_2_ NPs induced the secretion of TNF-α and IL-1β. The retinas were stained with antibodies against TNF-α and IL-1β and assessed by immunofluorescence staining. The levels of TNF-αand IL-1β were notably increased in the group injected with SiO_2_ NPs compared to the control. For example, the number of IL-1β-positive cells increased by 8- and 23-fold compared to the vehicle control at 1 and 7 d after SiO_2_ NP injection, respectively (Fig. 10A and C); the number of TNF-α-positive cells showed similar trends (Fig. 10B and D).

Taken together, the above results indicate that the SiO_2_ NPs caused retinal cell death and activated retinal inflammation.

### SiO_2_ NPs cause ROS overproduction

**Fig. 11:**
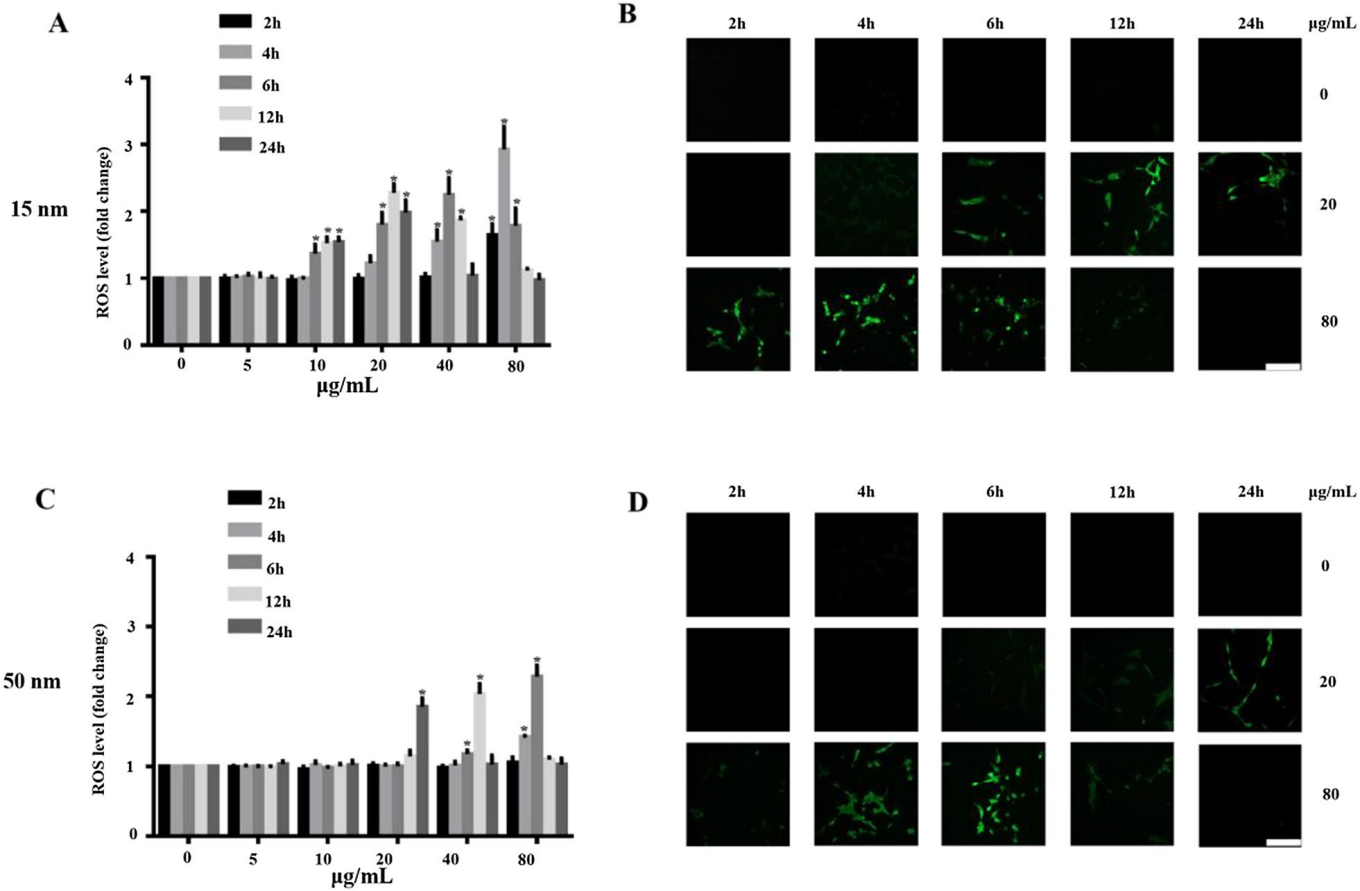
SiO_2_ NPs induce ROS generation. ROS levels were measured by H_2_DCF-DA staining at 2, 4, 6, 12 and 24 h after exposure to 15-nm (**A**) and 50-nm (**C**) SiO_2_ NPs at various concentrations (5–80 μg/ml). **B** and **D** The ROS levels were monitored by CLSM, showing that ROS level increased with time after exposure to SiO_2_ NPs at concentrations of 20 and 80 μg/mL. Data points are mean ± SD from three independent experiments with three samples per concentration. *p < 0.05 compared to the control. Scale bar: 100 μm

Driven by the in vitro and in vivo effects of SiO_2_ NPs on cell viability, morphology, mitochondrial dysfunction, apoptosis, and inflammation, we investigated the potential mechanisms underlying the retinal toxicity of SiO_2_ NPs. Previous studies demonstrated that one of the main toxicity mechanisms of NPs involves ROS generation [15, 23, 49]. Therefore, we first investigated whether the 15-and 50-nm SiO_2_ NPs induced oxidative stress. R28 cells were treated with SiO_2_ NPs at concentrations ranging from 5 to 80 μg/mL, and ROS production was monitored at 2, 4, 6, 12, and 24 h after treatment (Fig. 11). The SiO_2_ NPs were found to have size-, time-, and concentration-dependent effects on ROS generation. Compared with the control group, the ROS levels increased significantly within 2 h of treatment with 15-nm SiO_2_ NPs at 80 μg/ml. The ROS level continued to increase dramatically over time, reaching a maximum value (approximately 3 times that of the control group) at 4 h after treatment with 15-nm SiO_2_ NPs (Fig. 11A). Similarly, the ROS level increased significantly at 6 h after treatment with 50-nm SiO_2_ NPs (80 μg/mL); the maximum ROS level (2.3 times that of the control) occurred at 6 h after treatment (Fig. 11C). The ROS levels then decreased from 6 h to 12 and 24 h after treatment, presumably due to reduced cell growth (Fig. 3). To further verify the ROS generation results, we performed ROS fluorescence staining. R28 cells were treated with 15- and 50-nm SiO_2_ NPs at concentrations of 20 and 80 μg/mL. The CLSM images show that the ROS levels increased with incubation time (Fig. 11B and D). The increase in ROS level suggests that treatment with SiO_2_ NPs resulted in oxidative stress. Subsequent assays focused on the 15-nm SiO_2_ NPs.

### SiO_2_ NP-induced retinal toxicity is attenuated by ROS scavenging

**Fig. 12:**
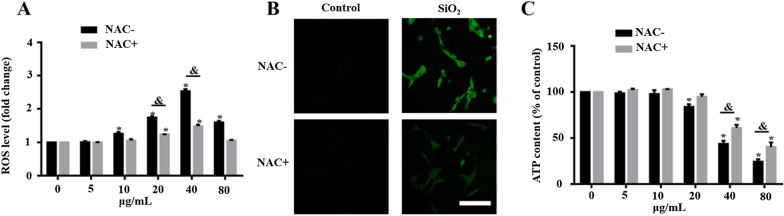
NAC attenuates SiO_2_ NP-induced cytotoxicity. **A** Intracellular ROS levels were measured at 6 h after SiO_2_ NP treatment with and without pretreatment with 10 mM NAC for 1 h. **B** ROS levels were monitored by CLSM, which showed that the ROS level increased at 6 h after exposure to SiO_2_ NPs at a concentration of 40 μg/mL (**C**) ATP content was evaluated at 12 h after treatment with SiO_2_ NPs with and without pretreatment with 10 mM NAC for 1 h. The data points are the mean ± SD from at least three independent experiments. *p < 0.05 compared to the vehicle control without NAC pretreatment; ^#^*p* < 0.05 compared to the vehicle control with NAC pretreatment; ^&^*p* < 0.05 between treatments with and without NAC pretreatment at the same concentration of SiO_2_ NPs

To further investigate the role of ROS generation in the retinal toxicity of SiO_2_ NPs, we used NAC, a ROS scavenger, to inhibit intracellular ROS generation. R28 cells were pretreated with NAC (10 mmol) for 1 h prior to treatment with SiO_2_ NPs (5–80 μg/ml) for 12 h. As shown in Fig. 12A, NAC significantly attenuated ROS induction. To further verify this result, we performed ROS fluorescence staining. The CLSM images show that pretreating cells with NAC before treatment with 40 μg/mL SiO_2_ NPs inhibited ROS production (Fig. 12B). Finally, the pretreatment of cells with NAC significantly decreased SiO_2_ NP-induced retinal toxicity in the R28 cells, as evidenced by the reduction in ATP content (Fig. 12C). These findings indicate that SiO_2_ NP-induced retinal toxicity was partially mediated by ROS generation.

## Discussion

The wide application of SiO_2_ NPs in the biomedical field has raised concerns regarding the safety of these NPs in humans and the environment. While the cytotoxicity of SiO_2_ NPs has been investigated by numerous scientists [3, 5, 35], most of these studies explored various SiO_2_ NP characteristics using a wide variety of in vitro models. Until now, no study has evaluated the retinal toxicity either in vitro or in vivo. In the present study, we explored the retinal toxicity of two types of SiO_2_ NPs with different sizes both in vitro and in vivo. We also investigated the potential mechanism underlying the retinal toxicity induced by SiO_2_ NPs.

Recently, SiO_2_ NPs have shown great potential in the treatment of ocular diseases [36–39]. Given the widespread use of SiO_2_ NPs to treat ocular diseases, the ocular toxicity of SiO_2_ NPs requires more attention from scientists and ophthalmologists. Park et al. reported that SiO_2_ NPs with sizes of 50, 100, and 150 nm did not induce significant cytotoxicity in cultured human corneal epithelial cells [40]. However, Chen et al. reported that SiO_2_ NPs led to cytotoxicity, ROS generation, and DNA damage in the human cornea [15]. SiO_2_ NPs can be used as intravitreal drug carriers [38, 41]; however, to the best of our knowledge, the retinal toxicity of SiO_2_ NPs has not been investigated before now. Therefore, we conducted both in vitro and in vivo experiments to evaluate the retinal toxicity of SiO_2_ NPs with sizes of 15 and 50 nm. The in vitro study used human R28 retinal precursor cells, which are expected to mimic in vivo responses.

We first evaluated the effect of SiO_2_ NP size on retinal toxicity. First, the particle morphology and average size were examined by SEM and TEM (Fig. 1). The two types of SiO_2_ NPs had sizes of 15 ± 5 and 50 ± 5 nm. The images of TEM showed the slight aggregation of SiO_2_ NPs. Since the toxicity of NPs depends on their phagocytosis by cells, the toxicity depends not only on their size and concentration, but also on their aggregate size and surface properties [50–52]. Hence, the hydrodynamic size distribution of NPs and zeta potential of both the NPs in distilled water, FBS-free medium, medium + 1%FBS, and medium + 10%FBS were further analyzed (Tables 1 and 2). The results indicated that DMEM + FBS promoted the aggregation of NPs. This result is similar with an earlier study [53], showing that buffered solutions promote protein adsorption onto NPs and particle agglomeration. By contrast, another study reported that exposure to FBS decreased the aggregate size of titanium dioxide (TiO_2_) nanoparticles, affecting the uptake and consequent effects of A549 and H1299 cells, however, this decreased of aggregation reduce the TiO_2_-induced cytotoxicity. Furthermore, it has been reported that 10% FBS in cell culture medium decreased the cytotoxicity of graphene oxide (GO) in A549 cells, and this decrease is due to the adsorption of GO by FBS [52]. Interestingly, an antibacterial study of graphene showed that in the graphene sheets-melatonin-bacterial suspension, aggregation of the sheets resulted in inactivation of *E. coli* bacteria. In our study, to avoid the affection of FBS and aggregation, the NPs in FBS-free culture medium were sonicated for 40 min in ice-bath before all treatments [54]. The 15 nm particles showed greater toxicity in R28 cells than the 50 nm particles (Figs. 3 and 4), consistent with previous studies on the effect of NP size on toxicity [1, 11, 55]. TEM imaging that both sizes of SiO_2_ NPs were taken up by R28 cells within 24 h and localized within the cytoplasm (Fig. 5). Importantly, the 15-nm SiO_2_ NPs were found in the mitochondria in addition to in the cytoplasm, which may explain why the 15-nm SiO_2_ NPs were more toxic than the 50-nm particles. Several studies have shown that nanomaterials including SiO_2_ NPs and graphene oxide nanowalls (GONWs) can damage cell membranes. For example, Bauer et al. [56] reported that SiO_2_ NPs were found on the cell membrane outside the cells after exposure for 2 h, and entered the cytoplasm within 24 h of incubation, which monitored by three-dimensional atomic force microscopy and fluorescence microscopy. Shinto et al*.* [57] found that cell membrane disruption induced by SiO_2_ NPs in different types of cells, including erythrocytes, lymphocytes, malignant melanocytes, and macrophages. In subsequent study, they found that interfacial serum proteins reduced the membranolysis [58]. An in vitro study using bacteria demonstrated that GONWs exhibited antibacterial activity due to their sharp edges that can interact with cell membranes [59]. In contrast, it is difficult to detect the damage to the cell membrane caused by SiO_2_ NPs by TEM in our study. Chen et al. [60] observed 70-nm SiO_2_ NPs in the nuclei of human epithelial HEp-2 cell; however, in the our study, the nuclear translocation of SiO_2_ NPs was not observed. The localization of NPs in cells can cause changes in cell morphology [61]. Thus, we evaluated the effects of the 15- and 50-nm SiO_2_ NPs on R28 cell morphology. Treatment with SiO_2_ NPs led to size-, time-, and concentration-dependent decreases in cell density and caused the cell shape to become ambiguous (Fig. 4).

As mentioned above, the 15-nm SiO_2_ NPs were more toxic than the 50-nm particles, consistent with previous studies finding that smaller particles tend to have higher cytotoxicity than larger particles. Thus, subsequent toxicity assays focused on the 15-nm SiO_2_ NPs. Mitochondria play a key role in cell survival and are the primary location of ATP production. Mitochondrial depolarization can lead to a decrease in ATP level. In this study, the SiO_2_ NPs induced mitochondrial dysfunction (Fig. 6). This finding is in good agreement with observations from previous studies [62, 63], where SiO_2_ NP-induced cellular damage was attributed to their mitochondrial dysfunction.

Because the concentration of SiO_2_ NPs used as a drug carrier was reported to be 100 μg/mL [38], we studied the in vivo retinal toxicity of SiO_2_ NPs at a concentration of 80 μg/mL, which was the highest concentration used in our in vitro experiments. First, cell death and TUNEL assays were performed. Consistent with the in vitro results, the SiO_2_ NPs showed time-dependent toxicity in the retina (Figs. 7 and 8). This is similar to an earlier study that reported the toxicity of SiO_2_ NPs (100 nm) and TiO_2_ NPs (100 nm) in R28 cells and retina [64]. As stated by the researchers of this study, there are some limitations, which are (1) only one size of NPs was studied; (2) apoptosis was only detected in in vivo studies, but functional relevance was not assessed. Notably, our study just makes up for their limitations. We compared the toxicity of 15-and 50-nm SiO_2_ NPs in two different retinal cell lines. More importantly, in our in vivo study, in addition to detecting apoptosis in various layers of the retina, we also detected damage to RGCs, which is very important for blindness. Furthermore, our results strongly indicate inflammatory cells were observed in the GCL (Figs. 7 and 8). Inflammation is the key factor in RGC damage. Our own results strengthen the idea that the injection of SiO_2_ NPs induced glial cell activation and RGC damage in a time-dependent manner (Fig. 9). In addition, our data indicate that the injection of SiO_2_ NPs induced the pro-inflammatory cytokines TNF-α and IL-1β (Fig. 10). Therefore, inflammation may be a factor in the retinal toxicity induced by SiO_2_ NPs.

Oxidative stress is the most studied factor in NP-induced toxicity because the small sizes and large surface areas of NPs are thought to generate ROS and induce oxidative stress [65]. After the cell is adversely stimulated, the mitochondria produce excess ROS due to an imbalance between ROS formation and the activity of the cellular antioxidant system. If the ROS cannot be completely degraded, the excess ROS will cause oxidative stress and induce cytotoxicity, leading to cell death [66]. Recent studies found that amorphous SiO_2_ led to ROS generation in MRC-5 human lung fibroblast cells [49] and MH-S macrophage cells [67]. Similar to this study, the present study demonstrated that 15- and 50-nm SiO_2_ NPs caused ROS generation in R28 cells in a size- and dose-dependent manner for the first time (Fig. 11). It is worth noting that, Akhavan et al. reported that Graphene/CuO_2_ nanoshuttles can release oxygen and capture respiratory electrons, and transfer them to oxygen nanobubbles, resulting in the generation of ROS and antibacterial effects. Unfortunately, SiO_2_ NPs can hardly release oxygen because they have only -oh chemical bonds and no loose oxygen to release (Fig. 2). Nonetheless, XPS revealed the presence of Si(III) in the nanoparticles (Fig. 2), which is reported to be one of the reasons for the generation of ROS [42]. However, it has been reported that SiO_2_ NPs can’t generate ROS in cell-free condition [68]. Our own results strengthen the endpoint that the impact of SiO_2_ NPs on cells is directly correlated with their cellular uptake (Fig. 5) as the NPs induce mitochondrial dysfunction (Fig. 6), which in the main course of ROS generation. Furthermore, the SiO_2_ NP-induced cytotoxicity in R28 cells was attenuated by pretreating the cells with NAC, an antioxidant (Fig. 12). This finding is in good agreement with a previous study in which cellular damage in vascular endothelial cells was attributed to the pro-oxidant effect of SiO_2_ NPs [4]. Our previous study has shown that ROS can induce DNA damage, which is one of the mechanisms involved in the cytotoxicity [32]. Interestingly, the similar mechanistic insight was also found in nanomaterial-induced toxicity. For example, it was found that carbon black (CB), single wall carbon nanotube, SiO_2_ and zinc dioxide (ZnO) nanoparticles can induce cytotoxicity, oxidative stress and DNA damage; however, their relationship has not been studied [69]. In subsequent studies of this line, several data and reports from in vitro studies suggest that DNA damage induced by SiO_2_ NPs is mediated by oxidative stress [70–72]. Besides SiO_2_, graphene nanoplatelets has also been reported to exhibit size-dependent ROS generation and genotoxicity in human stem cells [73]. It was found that cerium oxide NP-induced genotoxicity and apoptosis are mediated by ROS generation [74]. Consistent with this result, this mechanism can also be found in the toxicity induced by other metal NPs such as iron oxide [75], aluminum oxide [76], gold [77], and titanium dioxide NPs [78]. However, the research on the signaling pathways of SiO_2_ and other NP-induced toxicity is still limited, and additional studies are needed in the future.

## Conclusions

In summary, in the current study, both 15- and 50-nm SiO_2_ NPs induced cytotoxicity in R28 cells in vitro. Based on the decrease in ATP, LDH release, localization in the mitochondria, and ROS generation, the 15-nm SiO_2_ NPs were more toxic than the 50-nm NPs. The 15-nm SiO_2_ NPs also induced retinal toxicity and activated inflammatory response in vivo. ROS overproduction seems to play a critical role in the retinal toxicity induced by SiO_2_ NPs. The results provide new insights into the mechanism of SiO_2_ NP-induced retinal toxicity and improve our understanding of the potential hazards associated with the use of SiO_2_ NPs. It should be noted that this study did not consider the effects of SiO_2_ NPs on signaling pathways. Additional studies are needed to better understand the contribution of signaling pathways to SiO_2_ NP-induced retinal toxicity.

## Supplementary Information

**Additional file 1: Figure S1. **SiO_2_ NPs induce cytotoxicity in ARPE-19 cells. ARPE-19 cells were exposed to different concentrations (5–80 μg/mL) of SiO_2_ NPs for (A and C) 12 h and (B and D) 24 h before measurements of (A and B) ATP content and (C and D) LDH release. Data points represent the mean ± SD from three independent experiments with three samples per concentration in each experiment. *p < 0.05 compared to the control.

## Data Availability

All data generated or analyzed during this study are included in this published article.
