# Supplementary material for: Mechanistic study of silica nanoparticles on the size-dependent retinal toxicity in vitro and in vivo
Source: J Nanobiotechnology. 2022 Mar 19;20:146. doi: 10.1186/s12951-022-01326-8 (PMC8934510; doi:10.1186/s12951-022-01326-8)
Supplement: Supplementary file 1 — Additional file 1: Figure S1. SiO2 NPs induce cytotoxicity in ARPE-19 cells. ARPE-19 cells were exposed to different concentrations (5–80 μg/mL) of SiO2 NPs for (A and C) 12 h and (B and D) 24 h before measurements of (A and B) ATP content and (C and D) LDH release. Data points represent the mean ± SD from three independent experiments with three samples per concentration in each experiment. *p < 0.05 compared to the control. [file 12951_2022_1326_MOESM1_ESM.docx]

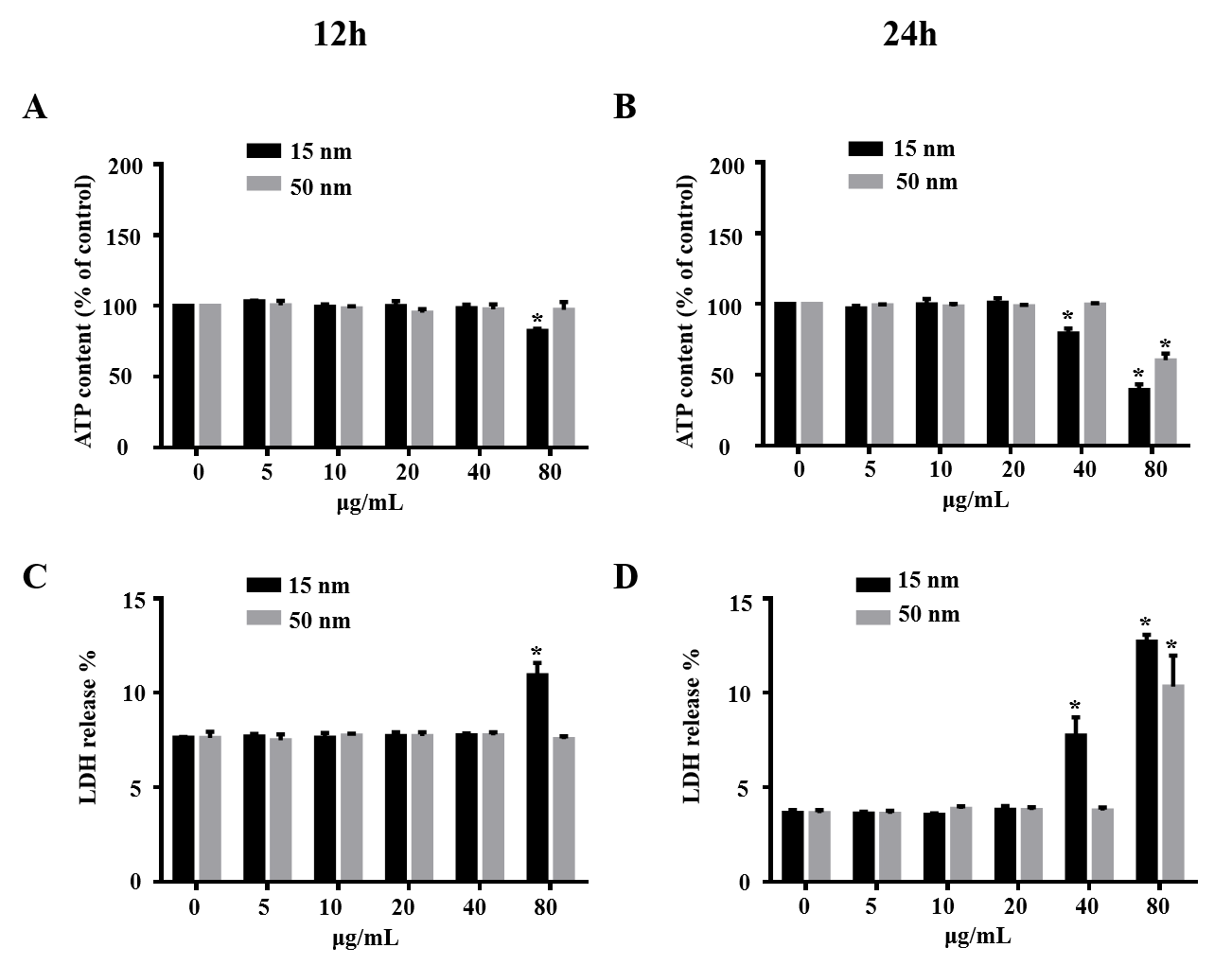
 Additional file 1: Figure S1. SiO_2_ NPs induce cytotoxicity in ARPE-19 cells. ARPE-19 cells were exposed to different concentrations (5–80 μg/mL) of SiO_2_ NPs for (A and C) 12 h and (B and D) 24 h before measurements of (A and B) ATP content and (C and D) LDH release. Data points represent the mean ± SD from three independent experiments with three samples per concentration in each experiment. **p* < 0.05 compared to the control.
